# Human antibodies against noncircumsporozoite proteins block *Plasmodium falciparum* parasite development in hepatocytes

**DOI:** 10.1172/jci.insight.153524

**Published:** 2022-03-22

**Authors:** Amanda Fabra-García, Annie S.P. Yang, Marije C. Behet, Zen Yap, Youri van Waardenburg, Swarnendu Kaviraj, Kjerstin Lanke, Geert-Jan van Gemert, Matthijs M. Jore, Teun Bousema, Robert W. Sauerwein

**Affiliations:** 1 Radboud University Medical Centre, Department of Medical Microbiology, Nijmegen, The Netherlands.; 2 Gennova Biopharmaceuticals Ltd., Pune, India.

**Keywords:** Infectious disease, Vaccines, Immunoglobulins, Malaria

## Abstract

Sporozoite-based approaches currently represent the most effective vaccine strategies for induction of sterile protection against *Plasmodium falciparum* (*Pf*) malaria. Clinical development of subunit vaccines is almost exclusively centered on the circum-sporozoite protein (CSP), an abundantly expressed protein on the sporozoite membrane. Anti-CSP antibodies are able to block sporozoite invasion and development in human hepatocytes and subsequently prevent clinical malaria. Here, we have investigated whether sporozoite-induced human antibodies with specificities different from CSP can reduce *Pf-*liver stage development. IgG preparations were obtained from 12 volunteers inoculated with a protective immunization regime of whole sporozoites under chloroquine prophylaxis. These IgGs were depleted for CSP specificity by affinity chromatography. Recovered non-CSP antibodies were tested for sporozoite membrane binding and for functional inhibition of sporozoite invasion of a human hepatoma cell line and hepatocytes both in vitro and in vivo. Postimmunization IgGs depleted for CS specificity of 9 of 12 donors recognized sporozoite surface antigens. Samples from 5 of 12 donors functionally reduced parasite-liver cell invasion or development using the hepatoma cell line HC-04 and FRG-huHep mice containing human liver cells. The combined data provide clear evidence that non-CSP proteins, as yet undefined, do represent antibody targets for functional immunity against *Pf* parasites responsible for malaria.

## Introduction

Malaria remains an enormous global challenge, with 229 million new cases and 409,000 deaths reported in 2019. Its causative agent is the protozoan parasite from the genus *Plasmodium*, of which *Plasmodium falciparum* (*Pf*) is the most virulent. Since 2015, the rate of progress to malaria eradication has slowed (1), evidencing the need to develop new and better tools.

*Plasmodium* infection is initiated when an infected *Anopheles* mosquito releases, on average, tens to hundreds of sporozoites into the skin during a blood meal (2, 3). Sporozoites are extracellular, motile parasite forms that glide and migrate through the dermis to eventually enter the circulation and reach the liver (4). During this journey of up to 2–3 hours, free sporozoites are vulnerable while exposed to the host immune system (3, 5). Once inside the liver, sporozoites traverse a number of cellular barriers before eventually invading a hepatocyte for asymptomatic multiplication and maturation (4). After 7 days, asexual parasite forms are released into the circulation, giving rise to clinical manifestations of malaria.

Most efforts to develop a *Pf*-malaria vaccine have been directed to sporozoite/liver stages, since they represent a bottleneck in the life cycle of the parasite and can prevent progression of blood-stage disease. Vaccine candidates are mostly centered on the circum-sporozoite protein (CSP) as the most abundant and immunogenic sporozoite surface antigen (6, 7). RTS,S/AS01 and R21/MM vaccines are the major and most advanced representatives (8, 9). CSP-specific mAbs are able to block *Pf* sporozoite infection of hepatocytes and prevent further parasite development in vitro and in animal models (10–12). A potent anti-CSP mAb showed protective efficacy in a phase I clinical trial with healthy volunteers (www.clinicaltrials.gov; NCT04206332) after controlled human malaria infection (13).

As an alternative approach, whole sporozoites attenuated by a variety of methods to ensure timely and complete developmental life-cycle arrest have shown to induce high levels of clinical protection (14). Efforts to define correlates of protection have been undertaken (15), but exact immune targets for sporozoite-induced protection remain elusive. The most efficient whole-sporozoite strategy is chemoprophylaxis and sporozoites (CPS) immunization, known as the CPS regime inducing > 90% sterile protection in malaria naive donors by relatively low numbers of sporozoites delivered by either mosquito bites or syringe (16–18). CPS-induced sporozoite–specific antibodies are functional, preventing liver stage development in vitro and in vivo in animal models (16, 19, 20). Although antibodies against CSP and other well-known candidates are induced after immunization (16), there are also many antibodies against uncharacterized hypothetical candidates identified by serological screening using a *Pf*-protein microarray or antigen libraries (21–23).

Using samples of CPS-immunized volunteers, we studied the functional ability of antibodies with specificities other than CSP to impair *Pf* sporozoite invasion of the hepatoma cell line HC-04 in vitro and FRG-huHep mice engrafted with human hepatocytes in vivo.

## Results

### Sporozoite-specific antibodies induced after CPS immunization.

**Figure 1:**
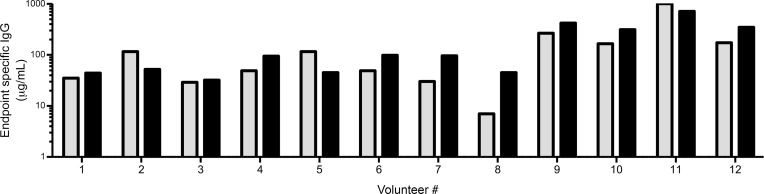
Induced sporozoite- and CSP-specific IgGs after immunization. Data represent specific IgG (μg/mL) corresponding to the lowest concentration that showed reactivity in ELISA compared with samples preimmunization (endpoint). Thresholds are set at the mean of preimmunization IgG of the 12 volunteers tested at 1 mg/mL + 3 SDs of the mean. OD_450_, 0.36 for the sporozoite ELISA; OD_450_, 0.59 for the CSP ELISA. Gray bars represent the reactivity of the IgGs against sporozoites, and black bars represent the reactivity against CSP.

Plasma was selected from 12 volunteers who received 3 immunizations with *Pf*NF54 sporozoites, where NF54 is the parasite strain used for immunization, under chemoprophylaxis with chloroquine (CPS immunization trial NCT02098590) (16). Sporozoite-specific antibodies were induced in the majority of the volunteers (11 of 12) with a substantial variation between individuals (Figure 1). The median increase in anti-sporozoite antibody titer after immunization was 14-fold (interquartile range [IQR], 5.8–31.7) higher as compared with baseline. Similarly, a strong interindividual variation was observed in the induced anti-CSP titer (Figure 1) with a median increase of 10.5-fold (IQR, 2.9–22.05) relative to baseline. Depletion of anti-CSP antibodies was performed by affinity chromatography with a column coated with full-length recombinant CSP. The depletion successfully removed recognition of CSP by ELISA, which was revealed when testing purified IgGs from all volunteers (Supplemental Figure 1; supplemental material available online with this article; https://doi.org/10.1172/jci.insight.153524DS1) and by Western blot with native sporozoite CSP protein (Supplemental Figure 2).

The overall reactivity in the 12 volunteers against sporozoite-lysate dropped significantly after the depletion (1-way ANOVA, *P* = 0.0001), and the signal, although not significant, remained higher than baseline. The combined data show that CSP is the prime antibody target of *Pf* sporozoites but that antibodies with non-CSP specificity are also induced after immunization with the CPS regime.

**Figure 2:**
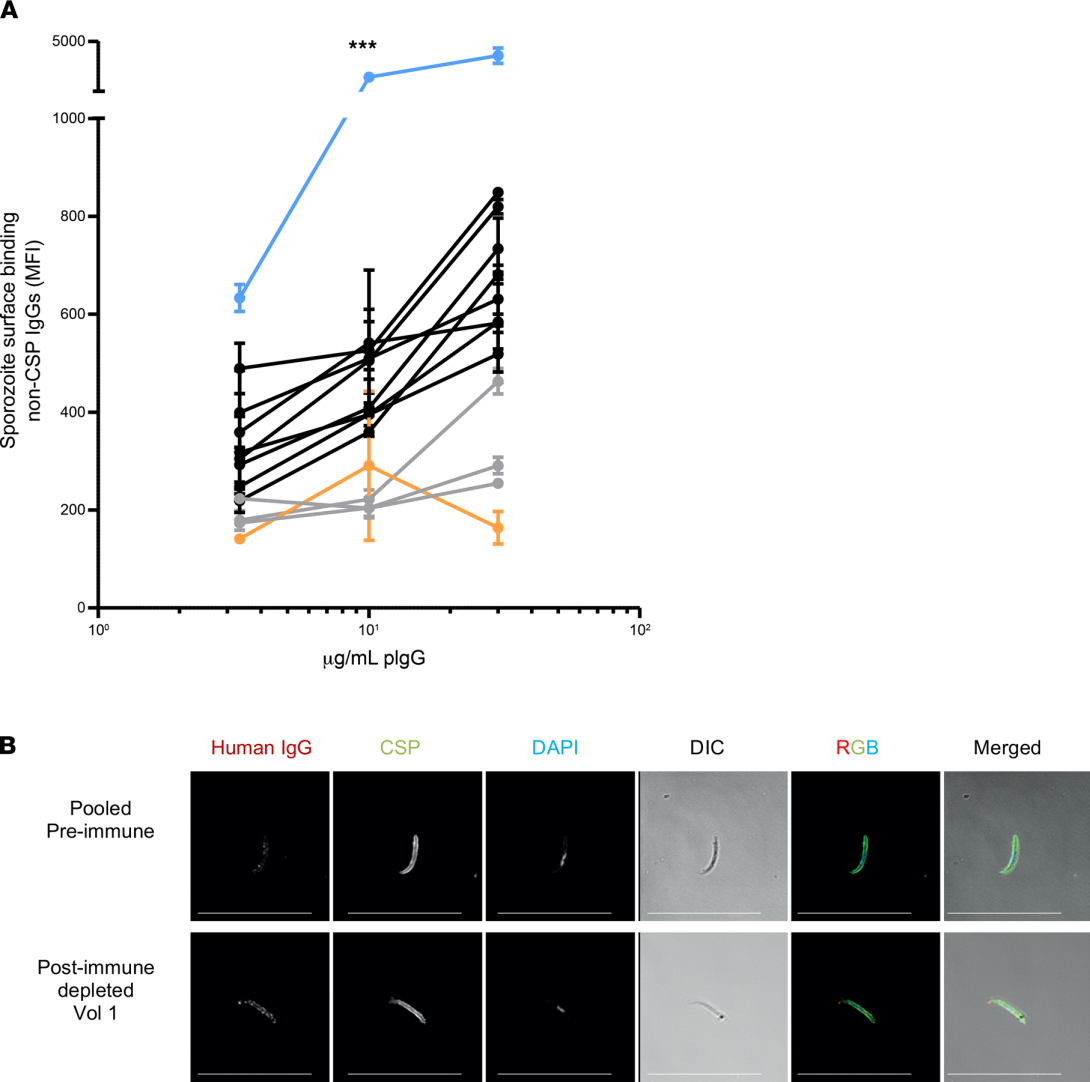
Sporozoite surface recognition before immunization and after depletion of CSP-specific antibodies. (**A**) Dose-dependent reactivity of sporozoite-specific polyclonal IgG with specificities different from CSP. Each line represents an individual volunteer, except for orange, which represented the reactivity of a pool of preimmunization samples (*n* = 12). Blue shows volunteer 1, with the strongest binding.Black shows volunteers with medium binding, and gray shows volunteers with low binding. Each sample was tested at 3 concentrations in duplicate, and a 2-way ANOVA was performed to determine dose response. There was an overall statistically significant dose response obtained when testing the IgGs at different concentrations (***P < 0.001). (**B**) Immunofluorescence with pool of preimmune samples and postimmunization depleted antibodies of volunteer 1 by microscopy. Red shows the reactivity of non-CSP antibodies, green shows CSP staining using mAb 3SP2, and blue shows nuclei staining with DAPI. Scale bar: 25 μm.

We next examined whether non-CSP-specific antibodies recognized proteins on the sporozoite membrane by flow cytometry (Supplemental Figure 3). A dose response in sporozoite surface staining was observed when testing the IgG at different concentrations (*P* < 0.0001) (Figure 2A). The AUC obtained after testing the IgGs at different concentrations was between 2 and 12 times higher than baseline in 9 of 12 volunteers (Table 1). Volunteers 10, 11, and 12 showed only weak or no surface recognition after CSP-specific antibody depletion. Overall, these data show that sporozoite membrane proteins different from CSP are able to induce specific antibodies in most volunteers after CPS immunization.

The anti-CSP depleted IgGs from volunteer 1, with the strongest surface recognition of sporozoites, showed an irregular and spotty pattern around the sporozoite membrane (Figure 2B).

We previously showed that CPS-immune sera also contain antibodies against a number of well-established targets including CSP, liver stage antigen 1 (LSA-1), exported protein 1 (EXP-1), thrombospondin-related anonymous protein (TRAP), a 19 kDa fragment of merozoite surface protein 1 (MSP-1), and the apical-membrane protein 1 (AMA-1) (21, 24). Supplemental Table 1 shows the induction profile of these antibodies in the 12 volunteers. All the targets except for AMA-1 were detectable by at least 1 volunteer — i.e., anti-CSP (*n* = 12 of 12), LSA-1 (*n* = 6 of 12), EXP-1 (*n* = 3 of 12), TRAP (*n* = 1 of 12), and MSP-1 (*n* = 4 of 12). Most of the volunteers (9 of 12) recognized at least 1 antigen, and 4 of 12 volunteers recognized multiple non-CSP antigens. While volunteer 1 showed the strongest surface recognition, no reactivity against any of the tested targets was detected, suggesting the presence of antibodies against sporozoite surface antigens different from the test panel.

### Inhibition of sporozoite invasion of the HC-04 cell line in vitro.

**Figure 3:**
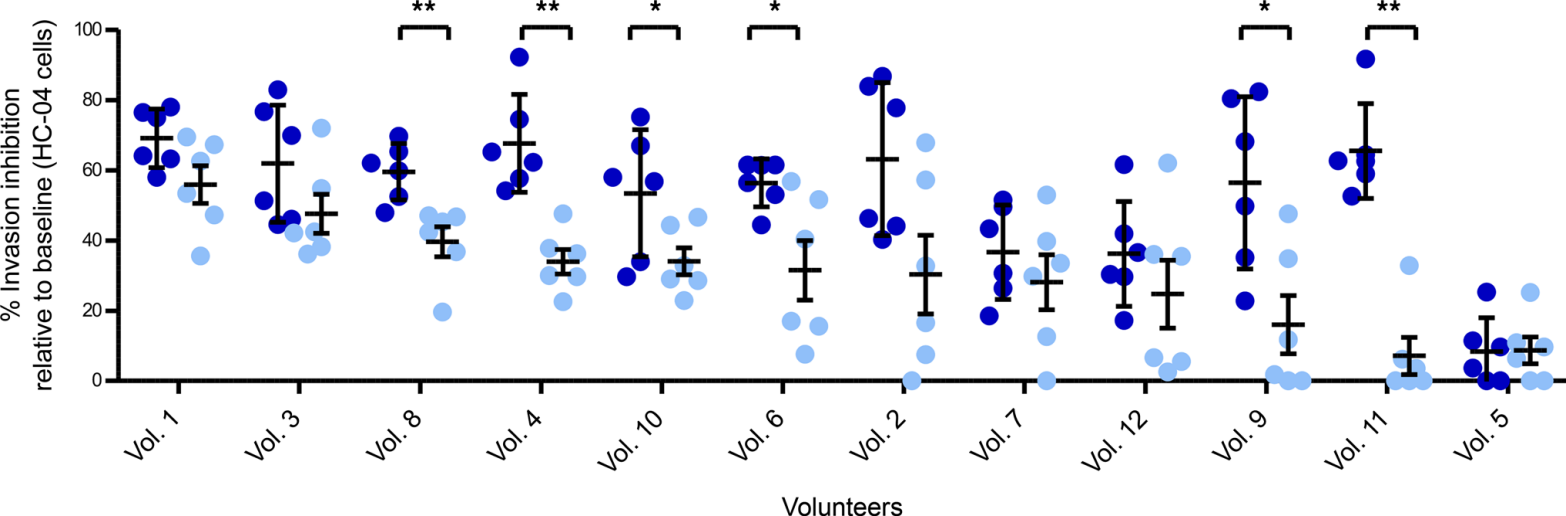
Hepatocyte Invasion inhibition of sporozoite in vitro. Postimmunization complete and depleted IgGs of 12 volunteers were tested at 4.5 mg/mL in human hepatoma cell line HC-04. Values represent the percentage of invasion inhibition activity. Data from postimmunization (dark blue bars) and IgGs depleted for CSP specificity (light blue bars) are represented as a percentage of invasion inhibition relative to preimmunization IgGs from the same volunteer (raw data in Supplemental Figure 4). The bars represent mean of 2 independent experiments performed with 3 technical replicates each. Data are shown as mean ± SD of 2 independent experiments. For each sample, 5000 HC-04 cells were analyzed by flow cytometry. *P* values are the result of Mann-Whitney *U* test; *P < 0.05; **P < 0.01.

The functional activity of postimmunization antibodies, complete or depleted for CSP specificity, was tested in a 3-hour sporozoite invasion assay with the HC-04 hepatoma cell line. Complete IgGs from 9 of 12 volunteers showed more than 50% mean invasion inhibition compared with baseline (Figure 3 and Table 2), which is in agreement with previous functional activity reported using primary hepatocytes (7 of 9 volunteers with strong reduction) (16).

After depletion of anti–CSP-specific antibodies, samples from 8 of 12 volunteers retained functional inhibitory activity up to 55% with the strongest activity by volunteer 1. In contrast, 4 of 12 donors showed weak or no activity (0%–25% activity) (Figure 3 and Table 2). The combined data indicate that CSP — but also other sporozoite proteins, probably coexpressed at the membrane — induce functional antibodies capable of preventing sporozoite invasion of HC-04 cells, in vitro.

### Inhibition of sporozoite invasion in humanized FRG-huHep mice in vivo.

To confirm our findings in an independent model, we next tested antibody functionality in vivo in FRG-huHep mice containing livers that are repopulated with human hepatocytes (25).

**Figure 4:**
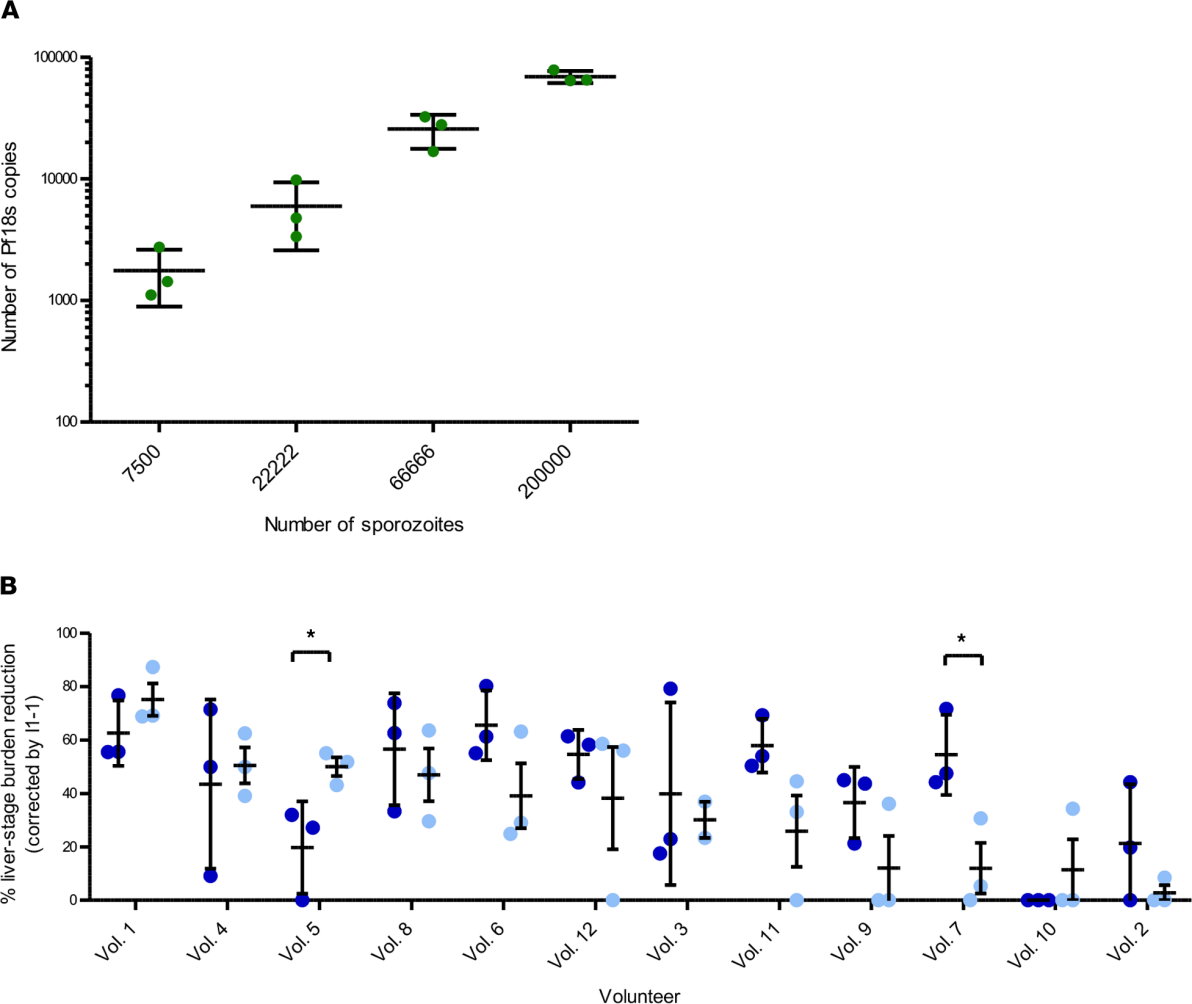
Parasite liver load in FRG-huHep humanized mice after infection with *Pf*NF54 sporozoites. (**A**) Graded numbers of *Pf*NF54 sporozoites were i.v. injected in groups of 3 FRG-huHep mice. The parasite load was quantified by the number of 18S copies in the qPCR. Bars represent mean, and data are shown as mean ± SD. (**B**) Pre- and postimmunization samples (complete or depleted for CSP specificity) were tested in groups of 3 mice for each time point. Each mouse received 6 mg of polyclonal IgG or 50 μg of 2A10 or PBS 24 hours before the challenge with 1 × 10^5^ *Pf* SPZ injected i.v. Genomic 18S copies were analyzed 6 days after i.v. infection of *Pf* sporozoites and normalized by the copies of PTGER (raw data in Supplemental Figure 5). Bars represent the percentage of liver burden reduction of complete IgG and CSP depleted IgG of 12 volunteers, corrected by baseline samples. Percentage of reduction of postimmunization samples complete (dark blue) and depleted (light blue). Data are shown as mean ± SD of 18S copies normalized by human PTGER, tested in duplicate for 3 mice. Anti-CSP mAb 2A10 used as positive control reduced the parasite liver burden by 10-fold compared with PBS. The mean number of copies in the control was 162,160 with SEM of 61,779. *P* values are the result of Mann-Whitney *U* test; *P < 0.05.

By testing serial numbers of injected sporozoites, we firstly determined an adequate liver load as defined by a Ct value of the *Pf*18S PCR (Figure 4A). A dosage of 1 × 10^5^ sporozoites was chosen as standard inoculum providing a Ct value between 24 and 22, allowing a sufficient signal for parasite quantification (>10,000 18S copies/mL).

Pre- and postimmunization samples (complete or anti–CSP depleted) were i.v. injected at 24 hours prior to sporozoite challenge. Complete postimmune IgG of 6 of 12 volunteers showed a strong (>50%) mean reduction of parasite burden compared with preimmune control IgGs, while moderate (25%–50% inhibition) was observed in 3 of 12 volunteers. The remaining 3 volunteers showed weak or no inhibitory activity (Figure 4B and Table 3). Interestingly, CSP depleted postimmune IgGs of 8 of 12 volunteers showed strong (*n* = 3) or moderate (*n* = 5) inhibition while the remaining 4 volunteers showed less than 25% inhibition (Figure 4B and Table 3). These data show that antibodies induced after CPS immunization with a non-CSP specificity are functional in this in vivo model.

## Discussion

In the present study, we show that antibodies against target antigens different from CSP are able to prevent sporozoite infection of a human hepatoma cell line in vitro and are able to reduce liver burden in humanized FRG-huHep mice in vivo. While CSP is the well-established and most advanced *Pf* vaccine to date (8, 9), we provide clear preclinical evidence that non-CSP proteins represent targets for functional preerythrocytic humoral immunity against *Pf*.

The dramatic decrease of sporozoite-specific antibody titers after anti-CSP depletion highlights the immune-dominance of CSP on the sporozoite membrane. It is estimated that 1 million copies of CSP are expressed at the sporozoite surface with more than 10 million antibody binding sites due to the repeats (6). Despite their lower expression at the membrane, it appears that non-CSP antigens are able to induce antibodies in the majority of our volunteers.

In the present study, depleted samples from 65% of the volunteers were able to reduce invasion in 1 functional assay, while 40% of the donors reduce parasite load in both liver assays. The discrepancy between assays may at least be partially explained by the parasite stage analyzed or the different mechanism of action and specificity of the antibodies. The HC-04 cell assay consists of a 3-hour period of incubation of hepatoma cells with parasites and, therefore, primarily focuses on early liver-stage development. Interestingly, here we show that the presence of antibodies targeting sporozoite membrane proteins correlates with invasion inhibition in vitro (Spearman’s ρ test, *P* = 0.038) but not in vivo (Spearman’s ρ test, *P* = 0.602) where invasion and maturation occur. This suggests that antibodies against membrane proteins may play roles in preventing sporozoite interaction with hepatocytes and, therefore, invasion and traversal, which is the focus of analysis of this in vitro model. Contrastingly, the FRG-huHep mice assay spans a 6-day period, and while, here, sporozoites were directly injected into the vein, parasites will be required to traverse the liver sinusoids to reach the underlying hepatocytes before final invasion. Since the antibody composition/specificities of each individual sera are not known, one could argue that the assay with FRG-huHep mice is closer to natural infection that requires the ability of the antibodies to block at 3 levels — invasion, traversal, and maturation — instead of only the first 2 needed for HC-04 cells assay. Therefore, not only sporozoite-specific, but also liver stage-specific antibodies may contribute in reduction of parasite burden in the liver. It is known that, after CPS-immunization, antibodies against liver stages are generated (21, 22). In the present study, volunteer 11 and volunteer 12 show weak or no sporozoite surface recognition, while they have antibodies against the liver stage proteins MSP-1 and EXP-1. Interestingly both volunteers show intermediate functional activity in vivo. It is tantalizing to speculate that antibodies targeting liver-stage antigens may be carried into the host hepatocytes and have an effect on maturation. However, this mechanism requires further investigation.

Although the mechanism of sporozoite infection is yet to be finalized, there is circumstantial evidence that TRAP (26), MAEBL (27), AMA-1 (27), and CelTos (28) may play a role in the invasion process. Therefore, antibodies against these proteins may functionally block invasion or traversal. While there is no evidence that AMA-1 or TRAP antibodies are able to impair *Pf* sporozoite invasion, mouse antibodies targeting MAEBL and CelTos have been proven functional (29, 30). So far, only antibodies against MAEBL have been identified after CPS immunization (21, 22). On the contrary, LSA-1, MSP-1, and EXP-1 are involved in liver stage development, merozoite formation, and maintenance of the parasitophorous vacuole. There is no evidence that antibodies against these targets functionally block sporozoite invasion, although this is yet to be confirmed. In our data set, we identified antibodies targeting TRAP, LSA-1, MSP-1, and EXP-1, but their potential functional role in preventing progression of liver stage development remains elusive. Remarkably, non-CSP antibody responses of volunteer 1 with the strongest sporozoite surface recognition and strongest functional responses in both assays remain negative in the test panel. This suggests a possible contribution of other uncharacterized sporozoite proteins on this strong inhibitory activity (21, 22). While an ELISA against *Pf*MAEBL or SEA1 was not available, we hypothesize that the observed antibody reactivity is not directed against *Pf*MAEBL; this protein has 2 AMA-1–like domains, and the sera show no recognition of AMA-1 (23, 31). However, it cannot be ruled out that the induced antibodies could bind to a different epitope not present in AMA-1. Clearly, downstream studies will be needed to further identify and define the antibody targets.

Our study is in agreement with earlier findings in rodent malaria. Mauduit and colleagues (32) showed that CSP-induced antibodies minimally contribute to the sterile protection using a CPS-like immunization regime. Therefore, sporozoite antigens other than CSP are able to induce functional responses. As the complexity and specificities of non-CSP antibodies are unknown, one may argue that 1 hypothetical non-CSP target may be as potent as CSP (as a single target) or that there may be multiple less potent targets responsible for the functional activity observed here. Delineation of composition and complexity of the specific antibody repertoire may include the selection of B cells from volunteers with strong non-CSP binding for in vitro stimulation to induce the production of specific antibodies. Once it is confirmed that the antibodies produced by the B cells recognize sporozoite proteins different than CSP, recombinant mAbs can be generated from the genetic material of the selected B cell (33). Mass spectrometry or microarrays can be used to identify mAb targets, while their functionality will have to be tested with hepatic cell lines and in in vivo models. The identified mAbs may further support structure-based vaccine identification, design, and clinical development as previously shown for CSP and RH5 vaccines (34, 35).

In conclusion, we identify the presence of sporozoite-specific non-CSP antibodies induced after CPS immunization with the capacity to impair liver stage development. Further characterization of the (fine) specificity of these functional non-CSP antibodies and their relative contribution to protection will contribute to a better understanding of antibody-mediated protection after natural infection or *Pf*-sporozoite immunization and will accelerate clinical development of protective vaccines and/or mAbs.

## Methods

### Study population and sample selection.

Plasma samples were obtained from 12 malaria-naive volunteers participating in a CPS immunization trial (NCT02098590) (16). Briefly, volunteers received 3 immunizations at 4-week intervals with *Pf* sporozoites delivered by the bites of mosquitos infected with NF54 sporozoites under chloroquine chemoprophylaxis. Samples were analyzed at baseline (preimmunization) and 1 day before challenge (18 weeks after the last immunization; postimmunization).

### In vitro cultures of Pf parasites.

*Pf* NF54 asexual and sexual blood stages were cultured in a semiautomated culture system as described before (36, 37). Sporozoites were produced by feeding *Anopheles stephensi* mosquitos (Sind-Kasur Nijmegen strain) using standard membrane feeding of cultured gametocytes, as previously described (38). Salivary glands were hand dissected, collected in Dutch modified RPMI 1640 media or DMEM F12 (Thermo Fisher Scientific), homogenized in a homemade glass grinder. Sporozoites were counted in a Bürker-Türk counting chamber using phase-contrast microscopy.

### Full-length recombinant PfCSP from Gennova.

As previously described (39), the synthetic nucleotide sequences encoding the translated protein sequence for PfCSP from the IND637HDDI (GenBank accession no. AAN87606) was commercially synthesized with codons optimized for maximizing expression of the heterologous gene in *E*. *coli*. This synthetic gene encoded the predicted full-length mature protein with a carboxy-terminal hexa-histidine tag, without the signal sequence and putative GPI anchor sequence.

Appropriate pET-24(+) vector (Novagen), derived from pBR332 plasmids, was used for cloning and expression of the PfCSP recombinant protein in *E*. *coli*. In pET-24(+) vectors, target genes were cloned under control of strong bacteriophage T7 transcription and translation signals, and expression is induced by providing a source of T7 RNA polymerase in the host cell. A vector-encoded carboxy-terminal hexa-histidine tag (in frame with the coding sequence of the target gene) was inserted to facilitate purification using immobilized-metal affinity-chromatography (IMAC). The pET-24(+) vector also encoded translation stop codons in all 3 reading frames following the cloning and the histidine-tag regions. Furthermore, the vector encodes an antibiotic-resistance gene allowing selection of transformant using growth medium supplemented with kanamycin. The drug resistance gene was cloned in the opposite orientation from the T7 promoter such that induction of the T7 promoter would not lead to overexpression of the kanamycin resistance gene product.

The synthetic *PfCSP* gene was cloned into the pET-24(+) vectors using appropriate restriction enzymes, minimizing the presence of vector-encoded residues and ensuring an appropriate open reading frame for expression of full-length proteins of interest. The resulting construct was used to transform nonexpression host cells such as DH5α *E*. *coli* cells for amplification. The recombinant plasmid DNA was then isolated using a Miniprep DNA isolation kit (Qiagen). The purified plasmid DNA was analyzed for the presence of the gene constructs using restriction enzyme digestion, and DNA-Seq would be carried out to check the full plasmid sequence. Verified plasmid constructs were used to transform the expression host BL21(DE3) *E*. *coli* cells (Novagen) for protein expression analysis.

For protein expression, the recombinant plasmid construct was used to transform BL2(DE3) *E*. *coli* cells. These host cells contain a chromosomal copy of the T7 RNA polymerase gene under the control of the *lacUV5* promoter, which is induced by the addition of a lactose analog such as isopropyl-β-D-thiogalactopyranoside (IPTG). IPTG induces production of T7 RNA polymerase allowing transcription of the target DNA in the plasmid. The BL21(DE3) strain is deficient in both *lon* and *ompT* proteases, thus improving stability of the recombinant protein expressed in these host cells.

Expression plasmid transformants were selected on kanamycin-containing medium. Well-separated single colonies were selected following growth on lysogeny broth (LB) agar plates containing kanamycin. Different parameters were tested for establishing the expression clone from the Research Plate as Master Cell Bank. These included tests for confirmation of cell morphology, Gram staining, confirmation of the host cell identity, plasmid restriction map analysis and DNA sequence verification, and verification of the plasmid stability and retention in the host cells.

### IgG purification and CSP-specific IgG depletion.

IgGs from plasma samples of 12 volunteers were purified by affinity chromatography, as previously described (40). The Igs were precipitated with a Saturated Ammonium Sulphate solution (Pierce, Thermo Fisher Scientific) and resuspended with PBS (Thermo Fisher Scientific). Subsequently, IgGs were purified with 5 mL Hi Trap protein G columns (GE Healthcare Life Science) bound to an automated liquid chromatography system, AKTA (GE Healthcare). PBS was used as binding buffer, and IgGs were eluted using a pH 2.8 amine-based buffer (Thermo Fisher Scientific).

CSP-specific IgGs were depleted as described elsewhere (40, 41). Briefly, an affinity chromatography column with covalently immobilized CSP was generated by coupling 1 mg of full-length (FL) recombinant (r) CSP protein (r*Pf*CSP FL Gennova) to NHS-activated Sepharose High Performance resin (GE Heathcare) according to manufacturer instructions. PBS was used as a binding buffer, and pH 2.8 amine-based buffer (Thermo Fisher Scientific) was used as elution buffer.

After purification and CSP depletion, IgGs were buffer exchanged to PBS using a centrifugal filter unit (Amicon Ultra-15, 30 kDa; MilliporeSigma), and total IgGs were quantified using NanoDrop 1000 (Thermo Fisher Scientific).

### Western blot with sporozoite lysates.

The reactivity of pools of purified IgGs before immunization (*n* = 10) and after immunization (*n* = 10), or those depleted (*n* = 6), was assessed in a Western blot on sporozoite lysates. To generate the lysates, 1 million NF54 sporozoites were incubated with 100 μL of lysis buffer (150 mM Nacl, 20 mM Tris-Hcl, 1% triton, 1 mM EDTA at pH 7.5 and 1× protease inhibition cocktail; Thermo Fisher Scientific) for 15 minutes on ice followed by a 10-minute centrifugation at 13,000*g* at 4°C. Protein lysate corresponding to 1 × 10^5^ sporozoites were loaded per well on a 4%–12% Bis-Tris Protein Gels. Proteins were transferred into a nitrocellulose membrane (Bio-Rad), and strips were made. After blocking for 1 h with 5% milk in PBST, the blots were incubated for 3 hours with 5 μg/mL of CIS43 (CSP-specific mAb; ref. 42) or pre- or postimmunization total or depleted purified IgGs tested at 5-point 1:2 dilution at a maximum concentration of 20 μg/mL. After 3 washes with PBST, we incubated samples with secondary antibody (goat anti–human IgG [H+L], HRP, 1:30,000, Thermo Fisher Scientific catalog A18805; polyclonal). Then, blots were washed 6 times with PBST and incubated with Clarity max ECL substrate (Bio-Rad). The imaging was performed in ImageQuant LAS4000 (Bio-Rad). The intensity of the bands was analyzed using ImageJ (NIH).

### ELISA.

Specific antibodies were analyzed using r*Pf*CSP FL or whole-sporozoite lysate, as described elsewhere (40, 41). Briefly, immunolon polystyrene flat-bottom 96-well plates (Thermo Fisher Scientific) were coated with 2 μg/mL of r*Pf*CSP FL or with a total of 3125 lysed *Pf*NF54 sporozoites per well. Purified IgGs of 12 volunteers were tested in duplicate at 8-point 1:3 dilution (complete postimmunization samples) or 4-point 1:3 dilution (pre- and postimmunization depleted samples) at maximum concentration of 1 mg/mL. The endpoint sporozoite- and CSP-specific IgG was calculated as the amount of IgG at the absorbance value obtained with the average of preimmunization IgGs + 3 SDs of the 12 volunteers tested at 1 mg/mL. Absorbance was measured at 450 nm using an iMark Microplate Absorbance Reader (Bio-Rad).

The reactivity of pooled purified IgGs obtained before immunization (*n* = 10, baseline signal) and of a pool of purified IgGs after immunization (*n* = 10 containing CSP-specific antibodies) or depleted for CSP specificity (*n* = 6) was evaluated in a r*Pf*CSP FL ELISA with a CSP-specific mAb (CIS43) as a positive control (42). Pools were tested in triplicate at 8-point 1:3 dilution at a maximum concentration of 1 mg/mL, as described above.

The specific antigen ELISAs were performed as previously described (24). Briefly, plates were coated with 2 μg/mL of either LSA-1, AMA-1, EXP-1, thrombospondin related anonymous protein (TRAP), or the 19 kDa C-terminal region of MSP-1_19_ (C-terminal 19 kDa region). Plasma samples were tested at a single dilution (1:100). The cutoff for seropositivity was analyzed for each antigen, and it was calculated as the mean + 3 SD of the averaged normalized OD 450 nm obtained with samples preimmunization of the 12 volunteers.

### Immunofluorescence.

*Pf*NF54 sporozoites were fixed with 4% paraformaldehyde for 10 minutes and washed 3 times in 1× PBS. After blocking with 3% BSA for 30 minutes, pre- or postimmunization polyclonal IgGs (pIgGs) were incubated (at a final concentration of 90 μg/mL in 3% BSA) for 1 hour at room temperature. Samples were washed 3 times with 1× PBS and incubated with goat anti–human Alexa 594 (Thermo Fisher Scientific) at a 1:200 dilution in 3% BSA for 1 hour at room temperature. After another 3 washes with 1× PBS, samples were incubated with DAPI and 3SP2-FITC (antibody against *Pf*CSP produced at Radboud University Medical Center; refs. 41, 43) at a 1:300 dilution in PBS for 1 hour. Samples were resuspended in PBS and spotted onto slides to air dry in the dark. Finally, VECTASHIELD (Vector Laboratories) was applied, and coverslips were mounted on top of the sample. Samples were imaged using a Zeiss LSM 880 confocal with Airyscan at 63× objective and 4× zoom.

### Immunofluorescence with flow cytometry readout.

*Pf*NF54 sporozoites were purified by gradient centrifugation using 17% Accudenz (Accurate Chemicals) in Milli-Q water (MilliporeSigma), as previously described (44). Subsequently, the sporozoites were incubated with 1 μM Syto61 Red Fluorescent Nucleic Acid staining (Invitrogen) for 30 minutes at 4°C. After 2 washes with PBS, sporozoites were centrifuged at 3220*g* for 5 minutes at 4°C and incubated in duplicate for 45 minutes at 4°C with a pool of preimmune IgGs or anti-CSP–depleted IgGs from each volunteer at 30, 10, and 3.3 μg/mL diluted in 3% BSA/PBS or with a CSP-specific antibody as a control (monoclonal anti-*Pf* CSP protein clone 2A10). After washing, goat anti–human IgG Alexa Fluor 488 (1:200 dilution, Invitrogen catalog A11013 polyclonal) was added for 30 minutes at 4°C. Finally, sporozoites were washed and fixed with 1% paraformaldehyde. Data corresponding to 5000 Syto 61^+^ cells (sporozoites) were acquired using a Gallios flow cytometer (Beckman Coulter) and analyzed with FlowJo software (version 10.0.8, Tree Star Inc.)

### Invasion inhibition assay using HC-04 cells.

*Pf* sporozoite liver cell invasion was tested in vitro with the hepatoma cell line HC-04 (MRA-965), as previously described (41). Ninety-six–well flat-bottom plates (Corning, Merck) were treated with rat tail collagen (MilliporeSigma; diluted 36× in Milli-Q water; for 3 hours at 37°C). Subsequently, HC-04 cells (5 × 10^4^ cells/well) were seeded for 16–24 hours at 37°C. *Pf*NF54 sporozoites dissected in DMEM-F12, were preincubated with 4.5 mg/mL before or after immunization (complete or depleted) pIgG and heat-inactivated malaria naive human serum for 30 minutes on ice or an anti-CSP mAb (2A10) (hybridoma cell line clone MRA-183, obtained from ATCC) at 100 μg/mL. Then, samples were added to each HC-04 well in triplicate, and plates were centrifuged at 1811*g* for 10 minutes at room temperature without brake and acceleration. After a 3-hour incubation at 37°C, plates were washed 3 times with PBS and incubated with trypsin (0.05% Trypsin-EDTA; Thermo Fisher Scientific) for 5 minutes. Trypsin was inactivated with 10% heat-inactivated human serum (HIHS) diluted in PBS, and cells were transferred to V-bottom, 96-well plates (Costar). HC-04 cells were stained with Fixable Viability Dye eFluor 780 (1:2000 dilution in PBS, eBioscience) for 30 minutes at 4°C. After a washing step with PBS, the cells were fixed and impermeabilized, using Foxp3 transcription factor staining buffer set (eBioscience). Subsequently, cells were stained with a mouse anti-CSP mAb (3SP2), labeled with Alexa Fluor 488 (1:600 dilution in perm buffer, mAb generated at Radboud University Medical Center; refs. 41, 43). Finally cells were fixed with 1% paraformaldehyde (Thermo Fisher Scientific), and data from 5000 HC-04 cells were acquired on a Gallios flow cytometer (Beckman Coulter) and analyzed with FlowJo software (version 10.0.8, Tree Star Inc.).

### FRG-huHep mice.

Male and female FRG-huHep mice (FRG/c57; 8–9 months old) with engrafted human hepatocytes from 3 different donors (HHM01008, HHF13022, and HHM19027) were purchased from Yecuris Corp. Repopulation of the mouse liver by the human hepatocytes was confirmed by measuring human albumin in the serum ranging between 4000 and 8000 μg/mL.

### In vivo infection of humanized mice.

To establish an appropriate parasite liver load, 12 female FRG-huHep mice (3 per group) were injected with graded numbers of *Pf*NF54 sporozoites (day 16 after infection) including 7500; 22,222; 66,666; or 200,000 parasites in 100 μL of Dutch modified RPMI 1640 media (Thermo Fisher Scientific). After 6 days, mice were sacrificed for collection of livers. Genomic DNA of the livers was extracted and quantified for *Pf*18S (parasite load).

In total, 114 FRG-huHep mice were grouped based on similar albumin levels into 12 test groups and 2 control groups. Test groups were divided into subgroups of 3 mice, each receiving either preimmune, complete postimmune, or CSP depleted postimmune IgGs from each volunteer. Infection control groups of 3 mice each received either PBS or 50 μg of 2A10 (mouse anti-CSP mAb) as a negative and positive control, respectively. Individual mice were injected with a standard dose of 6 mg pIgG in 200 μL (1× PBS) in the tail vein at 24 hours prior to sporozoite challenge. On the day of the sporozoite challenge, *Anopheles stephensi* mosquitoes infected with *Pf*NF54 (day 16) were dissected, and 1 × 10^5^ sporozoites in 100 μL of Dutch modified RPMI media (Thermo Fisher Scientific) were injected into the tail vein of each mouse. After 6 days, mice were sacrificed and the livers were collected. Genomic DNA was extracted as previously described (45) and quantified for *Pf*18S (parasite load) and human/mouse PTGER (humanization of liver) (46).

### Data availability.

All data underlying the results are available as part of the article, and no additional source data are required.

### Statistics.

Data were analyzed with GraphPad Prism 5.03. Dose-effect responses were analyzed with a 2-way ANOVA. Functional data were represented as a percentage relative to baseline samples, and it is shown as mean ± SD. One-way ANOVA was used to compare observation between 3 groups, and Mann-Whitney *U* test was used to compare observations between 2 groups. Correlations were analyzed with Spearman’s ρ.

### Study approval.

The study was approved by the Animal Ethics Committee of the Radboud University Medical Center (ADV103002016452). All participants provided written informed consent to be included in this study.

## Author contributions

This study was designed by RWS, AFG, ASPY, and MCB. Funding was obtained by RS. The experiments were performed by AFG, ASPY, ZY, YVW, KL, and GJVG. The data analysis was performed by AFG and RWS. The data were discussed by RS, AFG, ASPY, MMJ, TB, and SK. The first draft of the manuscript was written by AFG, ASPY, and RWS. The final draft of the manuscript was critically reviewed by MMJ and TB and commented on by all the authors.

## Supplementary Material

Supplemental data

## Figures and Tables

**Table 3:**
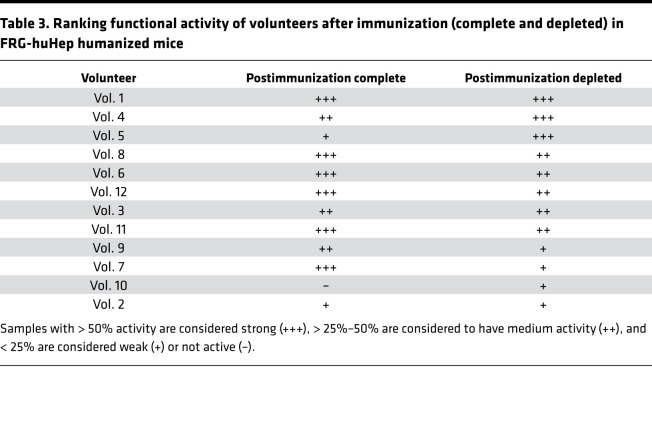
Ranking functional activity of volunteers after immunization (complete and depleted) in FRG-huHep humanized mice

**Table 2:**
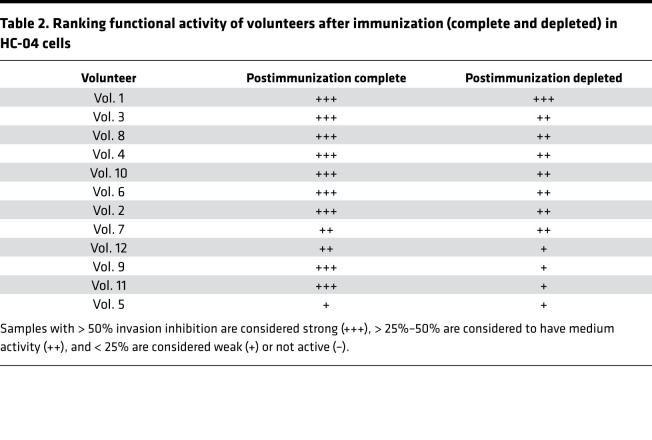
Ranking functional activity of volunteers after immunization (complete and depleted) in HC-04 cells

**Table 1:**
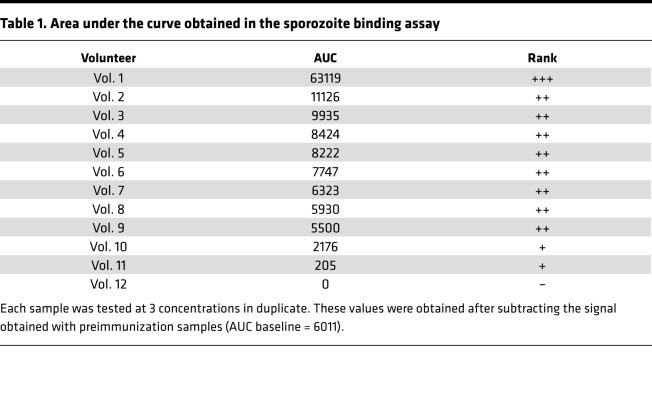
Area under the curve obtained in the sporozoite binding assay

## Version 1 02/15/2022

In-Press Preview

## Version 2 03/22/2022

Electronic publication
