# Supplementary material for: Human antibodies against noncircumsporozoite proteins block Plasmodium falciparum parasite development in hepatocytes
Source: JCI Insight. 2022 Mar 22;7(6):e153524. doi: 10.1172/jci.insight.153524 (PMC8986077; doi:10.1172/jci.insight.153524)
Supplement: Supplemental data [file jciinsight-7-153524-s026.pdf]

Supplementary Figure 1

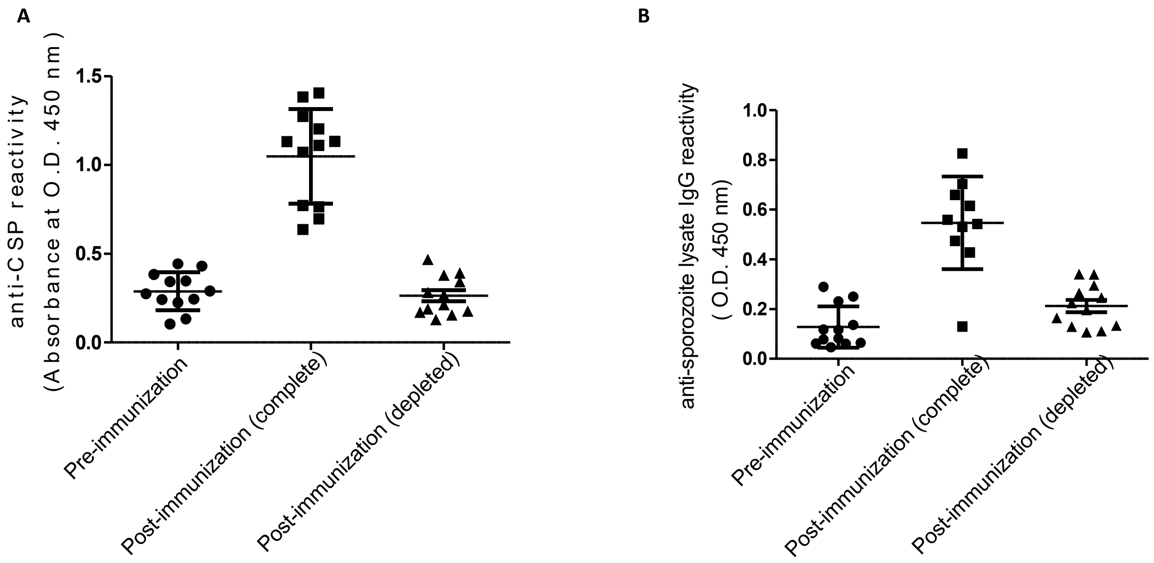

Supplementary Figure 1. Non-CSP antibody reactivity in ELISA. Samples from the 12 volunteers at pre-immunization (circles) and post-immunization complete (squares) and depleted (triangles) time points, tested at 1 mg/ml. A) CSP-specific ELISA. B) Sporozoite lysate-specific ELISA. One-way ANOVA showed that complete post-immunization samples are statistically significantly higher than the other tested samples ($p<0.001$).

Supplementary figure 2

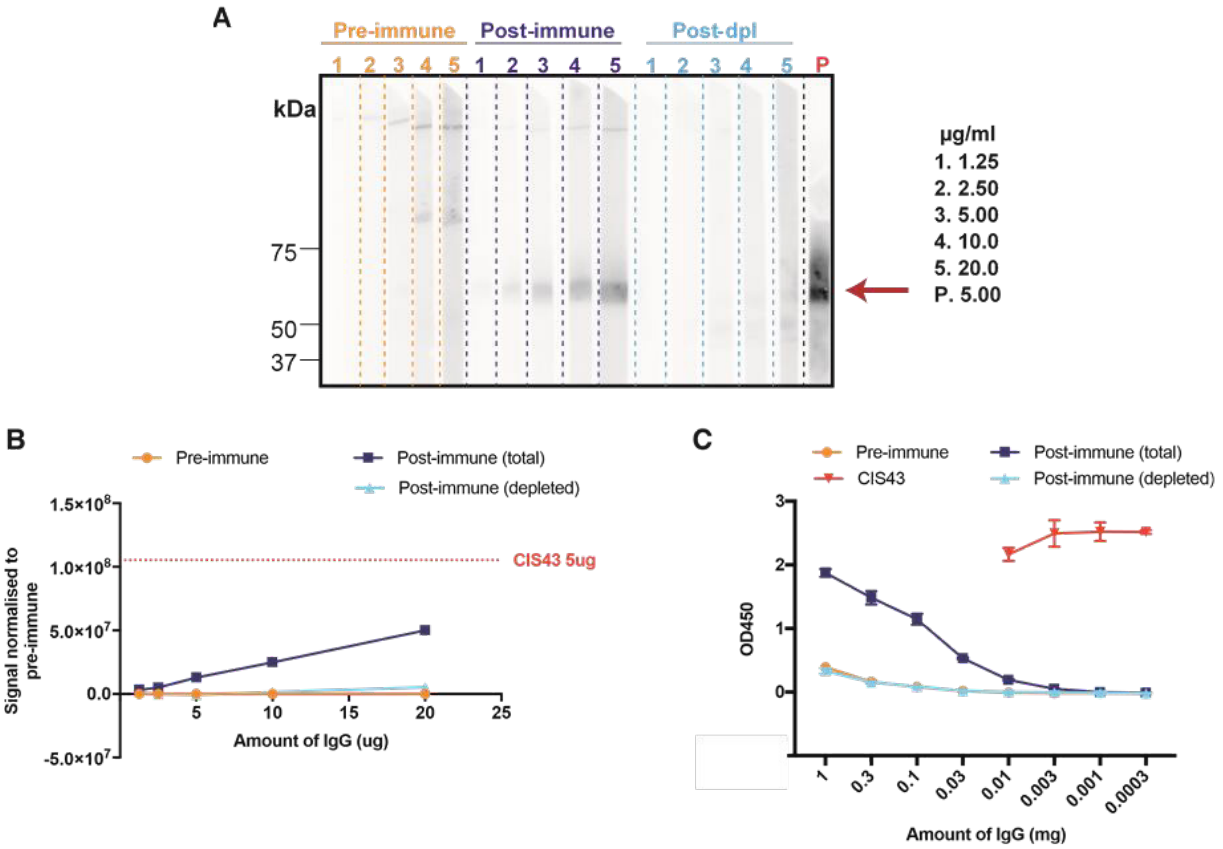

Supplementary figure 2: CSP-antibody depletion from pooled samples A) Sporozoite reactivity in western blot. Pools corresponding to pre-immune (orange) and post-immune complete (dark blue) and depleted (light blue) samples were tested at different concentrations on a western blot using 10^5 NF54 sporozoites per condition. P indicates the positive control (CIS43 a CSP-specific antibody). Size of the CSP band is indicated by the red arrow. B) Quantification of the anti-CSP signal. The intensity of the band corresponding to CSP was quantified using the software ImageJ. C) Reactivity in a CSP ELISA (Absorbance at OD_{450}). The same pooled samples were tested for its reactivity against the full-length recombinant CSP from Gennova.

Supplementary Figure 3

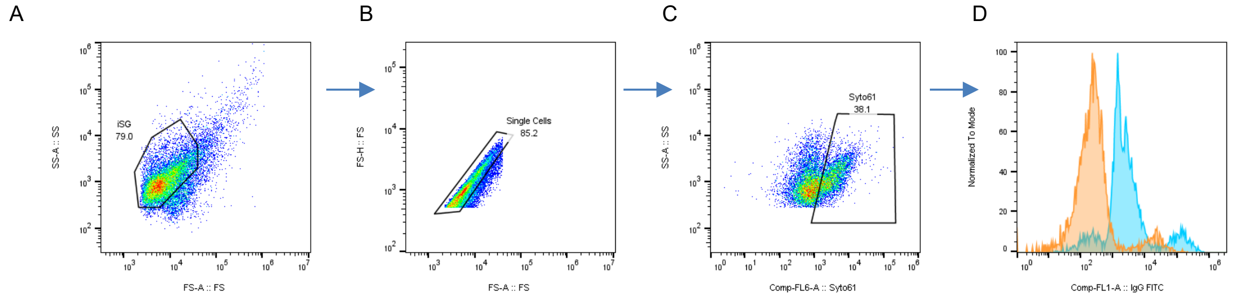

Supplementary Figure 3. Gating strategy followed for the sporozoite binding assay. Samples pre- and post-immunization (complete or depleted for CSP specificity) were tested for sporozoite surface protein recognition. The detection antibody was fluorescently labelled with Alexa Flour 488. Sporozoites from infected salivary glands were selected based on size and complexity (A) followed by the exclusion of aggregates (B). Single cells with nuclei staining (C) were selected to quantify the geometric mean of the IgG at the surface of sporozoites, represented as a histogram (D). The signal obtained with a pool of pre-immunization samples (orange), was set as negative and compared with post-immunization samples depleted for CSP specificity. in blue is represented the signal obtaind with depleted IgGs from volunteer 1 at 30 µg/ml.

Supplementary Figure 4

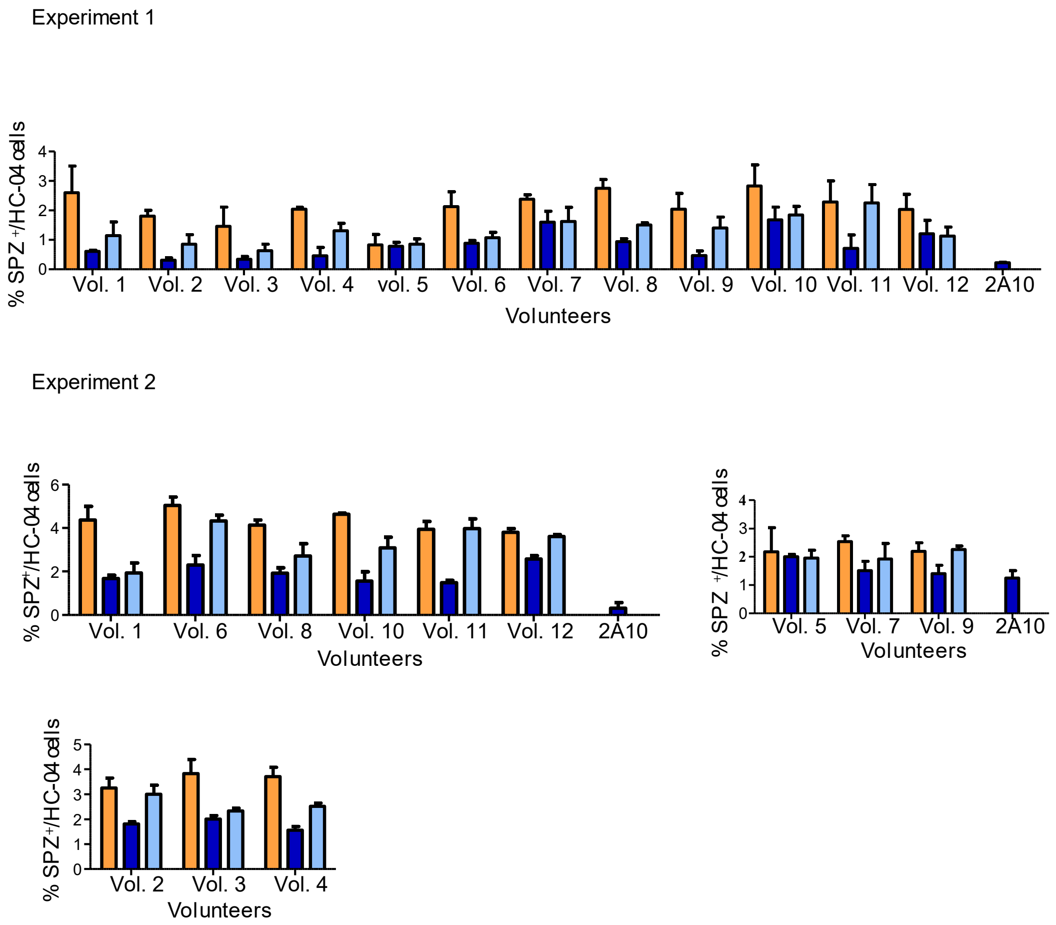

Supplementary Figure 4. Raw data invasion assay (HC-04 cells). Purified IgGs of 12 volunteers pre-immunization (orange), and post-immunization complete (dark blue) or depleted for CSP specificity (light blue) were tested in 2 independent experiments in triplicates. Data represent the percentage of HC-04 cells that are positive for intracellular sporozoite staining. Bars represent mean of technical replicates with corresponding standard deviation.

Supplementary Figure 5

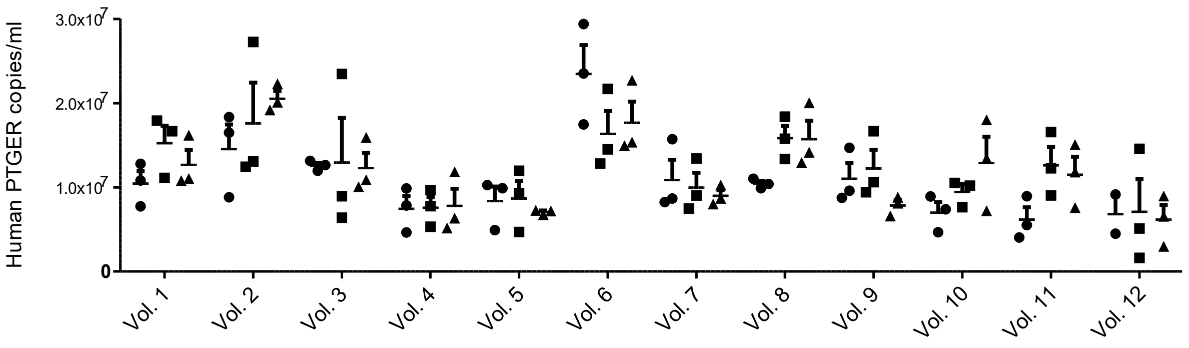

Supplementary Figure 5. Human PTGER copies in liver preparations from FRG-huHep mice. PTGER copies per ml was characterized by qPCR as previously described(46). 6 days after IV injection of sporozoites, mice that received pre- (circles) or post-immunization samples complete (squares) or depleted (triangles), were sacrificed and livers were processed.

Supplementary Table 1

VOL#	CSP	LSA-1	EXP-1	MSP-1	TRAP	AMA-1
1	+	-	-	-	-	-
2	+	-	-	-	-	-
3	+	+	-	+	-	-
4	+	+	-	-	-	-
5	+	+	-	-	-	-
6	+	+	-	-	+	-
7	+	-	-	-	-	-
8	+	+	-	-	-	-
9	+	+	+	+	-	-
10	+	-	+	+	-	-
11	+	-	+	-	-	-
12	+	-	-	+	-	-

Supplementary Table 1. Cross-stage antigen recognition of plasma after completed CPS-immunization. Pre- and post-immunization plasma samples were tested against specific sporozoite, liver and blood-stage antigens. Each sample was tested in duplicates. The cut off for seropositivity for each antigen was performed with the average signal obtained with pre-immunization samples + 3 standard deviations of the mean.
